# Characterisation of a putative glutamate 5‐kinase from *Leishmania donovani*

**DOI:** 10.1111/febs.14511

**Published:** 2018-05-27

**Authors:** Natasha Sienkiewicz, Han B. Ong, Alan H. Fairlamb

**Affiliations:** ^1^ Division of Biological Chemistry & Drug Discovery School of Life Sciences University of Dundee UK; ^2^ Present address: Cell Surface Signalling Laboratory Welcome Trust Sanger Institute Cambridge CB10 1SA UK

**Keywords:** biochemical pathway, drug target, inhibitors, *Leishmania*, proline biosynthesis

## Abstract

Previous metabolic studies have demonstrated that leishmania parasites are able to synthesise proline from glutamic acid and threonine from aspartic acid. The first committed step in both biosynthetic pathways involves an amino acid kinase, either a glutamate 5‐kinase (G5K; http://www.chem.qmul.ac.uk/iubmb/enzyme/EC2/7/2/11.html) or an aspartokinase (http://www.chem.qmul.ac.uk/iubmb/enzyme/EC2/7/2/4.html). Bioinformatic analysis of multiple leishmania genomes identifies a single amino acid‐kinase gene (LdBPK 262740.1) variously annotated as either a putative glutamate or aspartate kinase. To establish the catalytic function of this *Leishmania donovani* gene product, we have determined the physical and kinetic properties of the recombinant enzyme purified from *Escherichia coli*. The findings indicate that the enzyme is a bona fide G5K with no activity as an aspartokinase. Tetrameric G5K displays kinetic behaviour similar to its bacterial orthologues and is allosterically regulated by proline, the end product of the pathway. The structure‐activity relationships of proline analogues as inhibitors are broadly similar to the bacterial enzyme. However, unlike G5K from *E. coli*, leishmania G5K lacks a C‐terminal PUA (pseudouridine synthase and archaeosine transglycosylase) domain and does not undergo higher oligomerisation in the presence of proline. Gene replacement studies are suggestive, but not conclusive that G5K is essential.

**Enzymes:**

Glutamate 5‐kinase (http://www.chem.qmul.ac.uk/iubmb/enzyme/EC2/7/2/11.html); aspartokinase (http://www.chem.qmul.ac.uk/iubmb/enzyme/EC2/7/2/4.html).

**Abbreviations**
DDR: double drug‐resistant line
G5K: glutamate 5‐kinase
P5CS: Δ^1^–pyrroline‐5‐carboxylate synthase
PUA domain: pseudouridine synthase and archaeosine transglycosylase domain
^RC^WT: wild‐type leishmania‐overexpressing G5K
ROS: reactive oxygen species
SDR: single drug‐resistant line

## Introduction

Leishmania parasites are the causative agents of visceral, cutaneous and mucocutaneous leishmaniasis. The life‐threatening visceral form is responsible for 30 000 to 50 000 deaths per annum and the incidence of the disfiguring cutaneous form is estimated at 700 000 to 1 300 000 new cases per annum (https://www.dndi.org/diseases-projects/leishmaniasis/). Treatment options are limited: there are no efficacious vaccines and currently available drugs are expensive, difficult to administer and toxic 1, 2, 3. The disease is transmitted between mammalian hosts by the bite of a female sand fly. During their life cycle, the parasites undergo remarkable morphological and biochemical changes, ranging from motile flagellated promastigote forms living in the alkaline midgut of the sand‐fly vector to sessile amastigotes growing in the acidic parasitophorous vacuole of the host macrophage 4. Not only do leishmania have to adapt to environmental changes in pH, temperature and osmotic tension but also to changes in nutrient availability 5. In addition to intermittent blood feeds, the female sand fly also feeds on sugars (mainly sucrose) either from aphid honeydew or from plants. Thus, leishmania parasites live in a sugar‐ and amino acid‐rich environment in the insect midgut and preferentially use simple sugars as a source of energy. Under glucose‐limiting conditions, leishmania promastigotes can also use proline as an energy source 6, 7. Some bloodsucking insects utilise abundant proline in their haemolymph as an energy source for flight 8, 9, 10 where proline concentrations can be as high as 60 mm; however, nothing is known about proline levels in the case of sand flies. In contrast to the promastigote, leishmania amastigotes live in a glucose‐poor environment and utilise fatty acids for energy production and amino acids for gluconeogenesis 5, 11, 12, 13.

**Figure 1:**
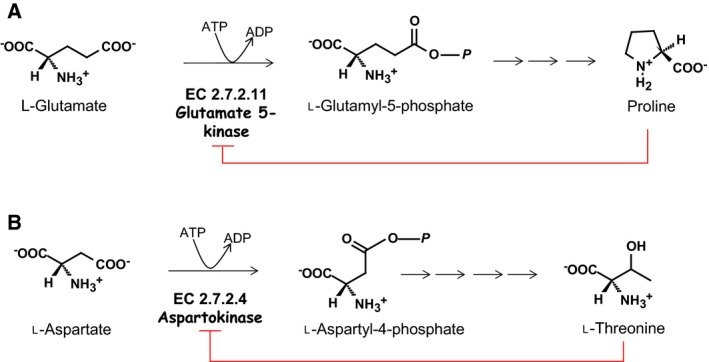
Proline and threonine biosynthetic pathways from glutamate and aspartate. *Leishmania* is predicted to synthesise proline from glutamate by the same pathway found in bacteria, comprising of three enzymes: γ‐glutamyl kinase (G5K, http://www.chem.qmul.ac.uk/iubmb/enzyme/EC2/7/2/11.html), γ‐glutamyl phosphate reductase (GPR, http://www.chem.qmul.ac.uk/iubmb/enzyme/EC1/2/1/41.html) and Δ^1^‐pyrroline‐5‐carboxylate reductase (P5C, http://www.chem.qmul.ac.uk/iubmb/enzyme/EC1/5/1/2.html). Biosynthesis of threonine is predicted to start with conversion of aspartate into l‐aspartyl‐4‐phosphate by aspartokinase (http://www.chem.qmul.ac.uk/iubmb/enzyme/EC2/7/2/4.html) followed by aspartate‐semialdehyde dehydrogenase (http://www.chem.qmul.ac.uk/iubmb/enzyme/EC1/2/1/11.html), homoserine dehydrogenase (http://www.chem.qmul.ac.uk/iubmb/enzyme/EC1/1/1/3.html), homoserine kinase (http://www.chem.qmul.ac.uk/iubmb/enzyme/EC2/7/1/39.html) and threonine synthase (http://www.chem.qmul.ac.uk/iubmb/enzyme/EC4/2/3/1.html).

Metabolic labelling studies with either [^14^C] or [^13^C] glucose indicate that label can be incorporated into glycine, serine, proline, or threonine in amino acid‐deficient medium 6, 14. [U^13^C] Aspartate is also readily incorporated into threonine suggesting the presence of the beta‐aspartate pathway to threonine 14. Glutamate and aspartate are of interest as the first committed step in the respective biosynthetic pathways involves an amino acid kinase, either glutamate 5‐kinase (G5K, http://www.chem.qmul.ac.uk/iubmb/enzyme/EC2/7/2/11.html) for proline synthesis, or aspartokinase (http://www.chem.qmul.ac.uk/iubmb/enzyme/EC2/7/2/4.html) for threonine synthesis (Fig. 1). Both of these pathways are absent in the related parasite *Trypanosoma brucei* where threonine constitutes a major source of acetate for lipid biosynthesis 15, 16, 17 and proline is a major energy source in insect procyclic forms 18, 19, 20. Although bioinformatic analyses of leishmania genomes have identified suitable candidate genes for all of the remaining pathway steps in the biosynthesis of proline and threonine 21, only one putative amino acid kinase can be identified (e.g. LinJ.26.2740, LmJF.26.2710, and LdBPK_262740.1). These genes are annotated as putative G5Ks in GeneDB, but their enzymatic function has not been characterised. In addition, they have also been proposed as possible aspartokinases 14, 22. The aim of this study was to characterise the putative G5K from *Leishmania donovani* and assess its possible role in proline and/or threonine biosynthesis.

## Results

### Sequence analysis of G5Ks

**Figure 2:**
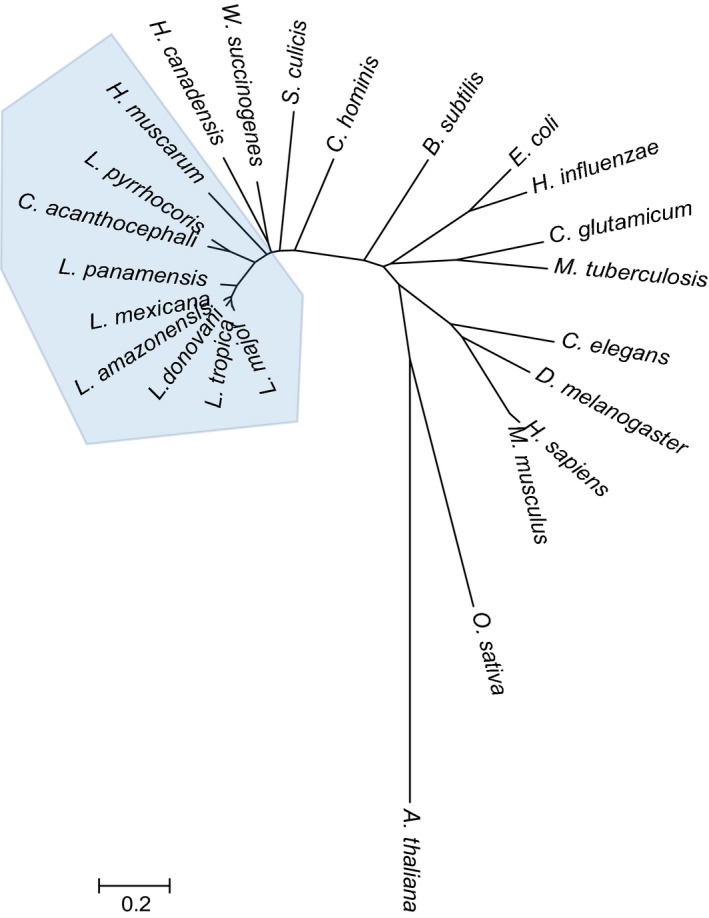
Phylogenetic relationship of G5K orthologues. The phylogenetic tree was constructed as described in the Experimental procedures. The full‐length sequence data were obtained from GenBank/EMBL databases under the following accession numbers: http://www.ncbi.nlm.nih.gov/protein/AEE32297.1 for *Arabidopsis thaliana* P5CS1; http://www.ncbi.nlm.nih.gov/protein/XP_015621839.1 for *Oryza sativa* P5CS; http://www.ncbi.nlm.nih.gov/protein/NP_001246877.1 for *Drosophila melanogaster* P5CS; http://www.ncbi.nlm.nih.gov/protein/CAA64224.1 for *Homo sapiens* P5CS; P5CS; http://www.ncbi.nlm.nih.gov/protein/NP_062672.2 for *Mus musculus* P5CS; http://www.ncbi.nlm.nih.gov/protein/CAC35828.2 for *Caenorhabditis elegans* P5CS; http://www.ncbi.nlm.nih.gov/protein/NP_216955.1 for *Mycobacterium tuberculosis* G5K; http://www.ncbi.nlm.nih.gov/protein/AAC44174.1 for *Corynebacterium glutamicum* G5K; http://www.ncbi.nlm.nih.gov/protein/AAC22560.1 for *Haemophilus influenzae* G5K; http://www.ncbi.nlm.nih.gov/protein/NP_414777.1 for *Escherichia coli* G5K; http://www.ncbi.nlm.nih.gov/protein/CAB13740.1 for *Bacillus subtilis* G5K; http://www.ncbi.nlm.nih.gov/protein/WP_012108545.1 for *Campylobacter hominis* G5K; http://www.ncbi.nlm.nih.gov/protein/EPY34138.1 for *Strigomonas culicis* G5K; http://www.ncbi.nlm.nih.gov/protein/WP_011138443.1 for *Wolinella succinogenes* G5K; http://www.ncbi.nlm.nih.gov/protein/EFR48368.1 *Helicobacter canadensis* G5K; http://www.ncbi.nlm.nih.gov/protein/AGT02536.1 for *Herpetomonasmuscarum* G5K; http://www.ncbi.nlm.nih.gov/protein/KPA74038.1 for *Leptomonas pyrrhocoris* G5K; http://www.ncbi.nlm.nih.gov/protein/AGT02656.1 for *Crithidia acanthocephali* G5K; http://www.ncbi.nlm.nih.gov/protein/AIN99380.1 for *Leishmania panamensis* G5K; http://www.ncbi.nlm.nih.gov/protein/AKK31239.1 for *Leishmania mexicana* G5K; http://www.ncbi.nlm.nih.gov/protein/AKK31245.1 for *Leishmania amazonensis* G5K; http://www.ncbi.nlm.nih.gov/protein/AKK31242.1 for *Leishmania donovani* G5K; http://www.ncbi.nlm.nih.gov/protein/AKK31236.1 for *Leishmania tropica* G5K; http://www.ncbi.nlm.nih.gov/protein/CAJ05678.1 for *Leishmania major* G5K. The trypanosomatid clade is highlighted in blue.

**Figure 3:**
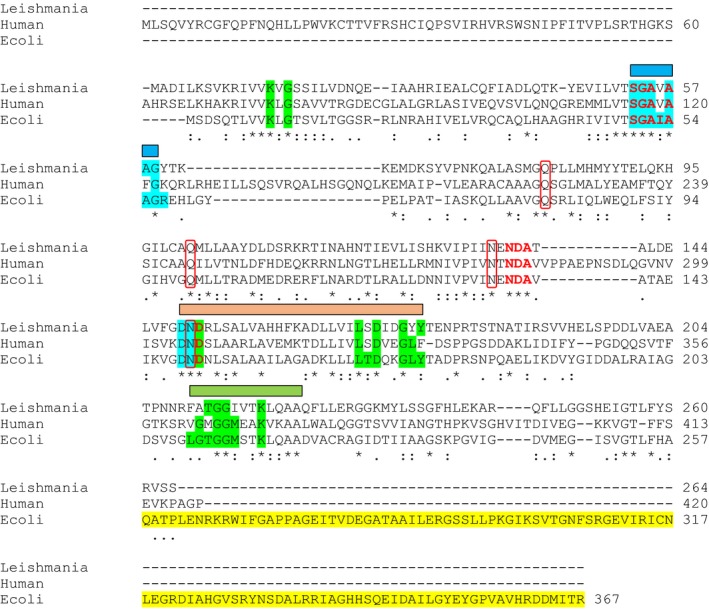
Multiple alignment and key functional residues in G5K orthologues. The amino acid sequence of *Leishmania donovani* G5K was compared to the human (excluding the C‐terminal PC5S domain) and *Escherichia coli* homologues. The amino acid sequences were aligned using muscle (http://www.ebi.ac.uk/Tools/msa/muscle/). Identical amino acid residues are highlighted*. Conserved residues which interact with the nucleotide (green), glutamate (red), interact with both (blue) and contain the PUA domain (yellow) are highlighted. Residues involved in linking the two catalytic centres of each dimer (red boxes). Binding motifs for ATP (blue rectangle), conserved G5K domain (green rectangle) and leucine zipper (peach rectangle) are also highlighted. Data from 23, 31, 80. All highlighted residues are identical in species causing mucocutaneous, cutaneous or visceral forms of leishmaniasis (*L. donovani* shows between 86 and 100% identity with *L. guyanensis, L. panamensis, L. braziliensis, L. mexicana, L. tropica, Leishmania major* and *Leishmania infantum*).

The construction of phylogenetic trees spanning both higher eukaryotes to lower prokaryotes (Fig. 2) was built based on their amino sequence and evolutionary distances were calculated by Poisson correction method within mega7 programme. *Leishmania* G5Ks (e.g. LmjF26.2710, LinJ.26.2740, and LdBPK_262740.1) are located on a clade closer to bacterial and lower eukaryotes compared to higher eukaryotes. A comparison of G5K sequences from *Escherichia coli*, human and *L. donovani* (Fig. 3) illustrates some shared homology in relation to residues interacting with nucleotides, glutamate as well as putative binding motifs for ATP, the conserved G5K domain and leucine zipper. Of note, only *E. coli* contains the C‐terminal PUA (pseudouridine synthase and archaeosine transglycosylase) domain, which is present in some bacteria but absent in the *Leishmania* equivalent. The PUA domain is potentially involved in RNA binding but its exact function is still unknown 8, 9. The situation is different in humans and other higher eukaryotes in that G5K is part of a bifunctional enzyme (Δ^1^–pyrroline‐5‐carboxylate synthase, P5CS). Here, the kinase domain at the N‐terminus is fused with a glutamate‐5‐semi‐aldehyde dehydrogenase (http://www.chem.qmul.ac.uk/iubmb/enzyme/EC1/2/1/41.html) domain at the C‐terminus. As with *Leishmania* the PUA domain is absent. It has been shown that in both bacteria and plants that proline biosynthesis is regulated by proline exerting feedback inhibition of G5K or the equivalent kinase domain of P5CS respectively 10.

### Cloning, expression and purification of recombinant LdG5K

**Figure 4:**
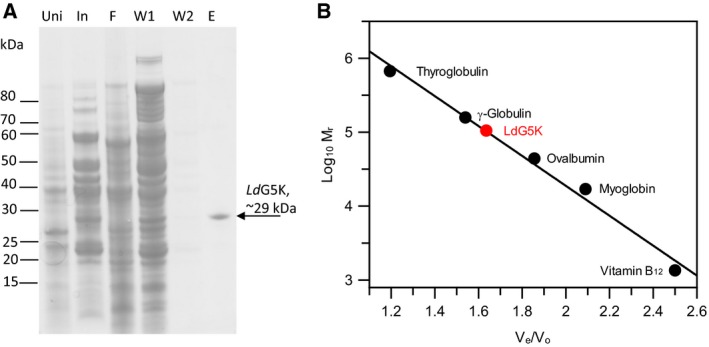
Purification and physical properties of recombinant LdG5K. (A) SDS/PAGE analysis of purification scheme using GST‐tagged LdG5K with on‐column cleavage resulting in the release of recombinant protein. Uni, uninduced sample; In, induced sample; F, flow through; W1 and W2, column wash 1 and 2; E, elution after on‐column cleavage with Prescission Protease. Molecular weight standards (kDa) highlighted on the left hand side of the gel. (B) Oligomeric structure of native LdG5K by size exclusion chromatography. Standard proteins and their molecular weights (Da) are as follows: Thyroglobulin (bovine), 670 000; γ globulin (bovine), 158 000; Ovalbumin (chicken), 44 000; Myoglobin (horse), 17 000; Vitamin B12, 1350. LdG5K, glutamate 5‐kinase.

The gene‐encoding LdG5K (0.792 kb) was amplified by PCR from *L. donovani* genomic DNA and cloned into a modified pGEX expression vector. After purification and on‐column proteolytic cleavage of the GST tag a single band was released (yield ~ 1 mg·L^−1^ of culture) with a M_r_ of ~ 29 kDa by SDS/PAGE (Fig. 4A). The theoretical Mr was calculated from the amino acid sequence to be 29.033 kDa. Confirmation was undertaken by tryptic analysis of the isolated protein determined by MALDI‐TOF mass spectrometry with 91% coverage and a mass of 29.10 kDa. The molecular weight of the native LdG5K was determined by size exclusion to be approximately 105 kDa consistent with a tetrameric quaternary structure (Fig. 4B).

### Biochemical characterisation and determination of the kinetic parameters

**Figure 5:**
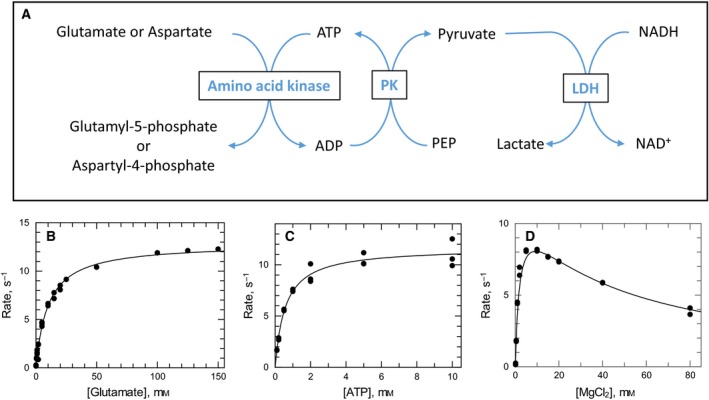
LdG5K activity relationship with varying substrates. In the standard coupled assay monitoring the oxidation of NADH (A), the enzyme concentration was fixed at 0.75 μg with varying concentrations of the following: (B) l‐glutamate range 0–150 mm with fixed 5 mm ATP and 5 mm MgCl_2_ concentrations; (C) ATP range 0–10 mm with fixed 5 mm MgCl_2,_ and Na Glutamate, 150 mm concentration; (D) MgCl_2_ range 0–80 mm with fixed 5 mm ATP and 150 mm Na Glutamate concentration.

Previous enzymatic activity assays have used fixed time‐point assays measuring product formation, either ADP 23 or γ‐glutamyl phosphate converted into γ‐glutamyl hydroxamate 24. A spectrophotometric assay previously described for trypanothione synthetase activity 25 was adapted to follow LdG5K activity with glutamate, aspartate or other amino acid analogues. The assay measured ADP formation by coupling the reaction to pyruvate kinase/lactate dehydrogenase and monitoring oxidation of NADH at 340 nm (Fig. 5A). The kinase exhibited typical hyperbolic kinetics with respect to l‐glutamate and ATP (Fig. 5B,C) with *k*_cat_ and *K*_m_ determined to be: 12.8 ± 0.3 s^−1^ and 10 ± 0.7 mm; 11.7 ± 0.3 s^−1^ and 0.6 ± 0.07 mm, respectively. Activation of G5K by Mg^2+^ shows high substrate inhibition with *k*_cat_ 11.6 ± 0.8 s^−1^, *K*_a_ 2.0 ± 0.4 mm and *K*_i_ of 43 ± 7 mm (Fig. 5D). These parameters are broadly similar to those for bacterial glutamate kinases (Table 1). Several analogues of ATP and l‐glutamate were tested as alternative substrates. GTP, CTP or UTP did not replace ATP as phosphate donor as reported for G5K from *Thermotoga maritima* 26. Glutaric acid showed 29% of the activity with glutamate as substrate, whereas aminovaleric acid and 2‐amino‐4‐phosphonobutyric acid were inactive. No activity whatsoever could be detected over a range of aspartate concentrations (1–200 mm). l‐citrulline and d,l‐ornithine at 1 mm concentration did not inhibit the enzymatic conversion of l‐glutamate. These data indicate that the enzyme is a G5K, with no functional activity as an aspartokinase.

**Table 1:** Comparison of G5K kinetic parameters with other species

Substrate/Metal ion	Parameter	Units	*Leishmania donovani*	*Escherichia coli* a		*Thermotoga maritima* b
				WT	G5K domain	
ATP	$K_{m}^{\mathrm{app}}$	mm	0.6 ± 0.07	1.6	1.6	3.3
	*k*_cat_	s^−1^	11.7 ± 0.3	85.6	33.9	52
	*k*_cat_/$K_{m}^{\mathrm{app}}$	m ^−1^·s^−1^	19 500	53 600	21 200	15 800
Glutamate	$K_{m}^{\mathrm{app}}$	mm	10 ± 0.7	91	227	23
	*k*_cat_	s^−1^	12.8 ± 0.3	109	48.8	51
	*k*_cat_/$K_{m}^{\mathrm{app}}$	m ^−1^·s^−1^	1280	1200	215	2200
Magnesium	$K_{a}^{\mathrm{Mg}}$	mm	2.0 ± 0.4	4.3	0.11	–
	$K_{i}^{\mathrm{Mg}}$	mm	43 ± 7	~ 15	4.8	–
	*k*_cat_	s^−1^	11.6 ± 0.8	77.2	21.7	–

The activity of LdG5K on varying concentrations of ATP, glutamate and free magnesium was determined by Pyruvate Kinase/Lactate Dehydrogenase coupling system. Free Mg was fitted to high substrate inhibition equation as described in the Experimental procedures. Data calculated from ^a^Pérez‐Arellano *et al*. 81 for the full‐length protein (WT) and for the protein lacking the PUA domain (G5K domain) and ^b^Pérez‐Arellano and Cervera 26.

### Effect of proline on LdG5K activity and its oligomeric state

**Figure 6:**
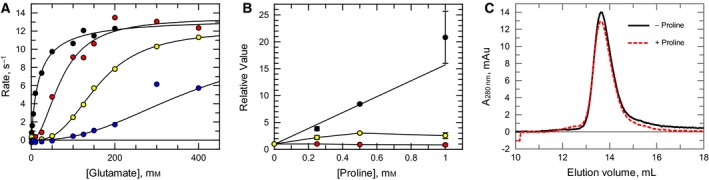
Effect of l‐proline on kinetic behaviour of LdG5K with l‐glutamate and influence on the oligomeric state. (A) The effect of varying l‐glutamate concentration on LdG5K activity in the absence (black) or presence of fixed concentrations (mm) of l‐proline (0.25, red; 0.5 yellow; and 1, blue). (B) Effect of proline on kinetic parameters: *S*_0.5_ (black); *k*_cat_ (red); Hill slope (yellow). Values (±standard error of the fit to the Hill equation) are expressed relative to parameters in the absence of proline (*S*_0.5_ 19.0 ± 2.9 mm;* k*_cat_ 13.5 ± 0.6 s^−1^; Hill slope 0.94 ± 0.09). (C) Elution profiles of LdG5K incubated in the presence (black line) and absence (red dotted line) of 10 mm l‐proline.

In the absence of proline, the substrate glutamate obeys hyperbolic kinetics (Fig. 5). However, the kinetic behaviour with glutamate as variable substrate becomes sigmoidal in the presence of increasing concentrations of proline (Fig. 6A). Relative to the kinetic parameters in the absence of proline, Hill slope increases to a maximum of 3.0, *S*_0.5_ increases linearly, whereas *V*_max_ decreases with increasing proline concentrations (Fig. 6B). This behaviour is similar to that reported for *E. coli* G5K 27. The effect of excess proline (10 mm) on the oligomeric state of LdG5K was examined as studies undertaken for the *E. coli* enzyme resulted in aggregation of tetramers into decamers 23. This was not the case for LdG5K, with no change in the oligomeric state being detected by size exclusion (Fig. 6C). This supports the conclusion that higher oligomerisation of *E. coli* G5K requires a PUA domain and that allosteric kinetic behaviour is conferred solely by the G5K domain 23.

### Inhibition of LdG5K by proline analogues

A panel of l‐proline analogues were surveyed for their inhibitory activity against LdG5K at a fixed concentration of 1 mm. Of these, d‐proline was inactive while l‐proline and three other compounds inhibited activity by more than 30% (Table 2). l‐proline was the most effective with an IC_50_ value of 0.39 ± 0.08 mm, followed by 3,4‐dehydro‐l‐proline, l‐azetidine‐2‐carboxylic acid and l‐4‐thiazolidine carboxylic acid. Methylation of either the α‐carbon or the imino nitrogen or esterification of the α‐carboxylate abolished the inhibitory activity of l‐proline. Similarly expansion of the pyrrolidine ring to a piperidine or various substitutions on the pyrrolidine ring were not tolerated (Table 2).

**Table 2:** Inhibition of recombinant LdG5K by proline analogues compared with *Escherichia coli* G5K. IC_50_ values where measured if LdG5K activity was < 70% in the presence of 1 mm proline analogue. The IC_50_ values were measured as described in the Experimental procedures by measuring the enzyme velocity as a function of inhibitor concentration and the data were fitted using a four‐parameter IC_50_ equation. NT, not tested

Proline analogue		LdG5K activity (%) with 1 mm	LdG5K IC_50_ (mm)	EcG5K IC_50_ (mm)^a^
l‐proline	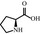	4	0.39 ± 0.08	0.15
d‐proline	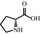	103	–	NT
3,4‐dehydro‐d,l‐proline	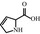	9	0.49 ± 0.02	0.16
l‐azetidine‐2‐carboxylic acid	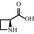	25	0.73 ± 0.07	1.38
l‐4‐thioproline	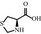	66	1.8 ± 0.2	1.0
l‐pipecolinic acid	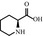	100	–	NT
d‐pipecolinic acid	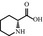	100	–	NT
*N*‐methyl‐l‐proline	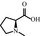	100	–	NT
l‐proline methyl ester	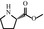	100	–	3.5
α‐methyl‐l‐proline	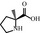	82	–	4.7
*Cis*‐3‐hydroxy‐l‐proline	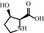	100	–	NT
*Cis*‐4‐hydroxy‐l‐proline	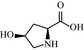	87	–	1.22
*Trans*‐4‐hydroxy‐l‐proline	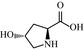	89	–	22.6
*N*‐acetyl‐l‐proline	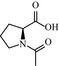	93	–	> 100
l‐trans‐pyrrolidine‐2,4‐dicarboxylic acid	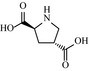	84	–	NT
l‐prolinamide	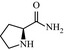	96	–	NT
Trans‐1‐acetyl‐4‐hydroxy‐l‐proline	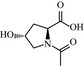	100	–	NT

^a^Data from Pérez‐Arellano *et al*. 23.

### Genotypic analysis of WT and KO cells

**Figure 7:**
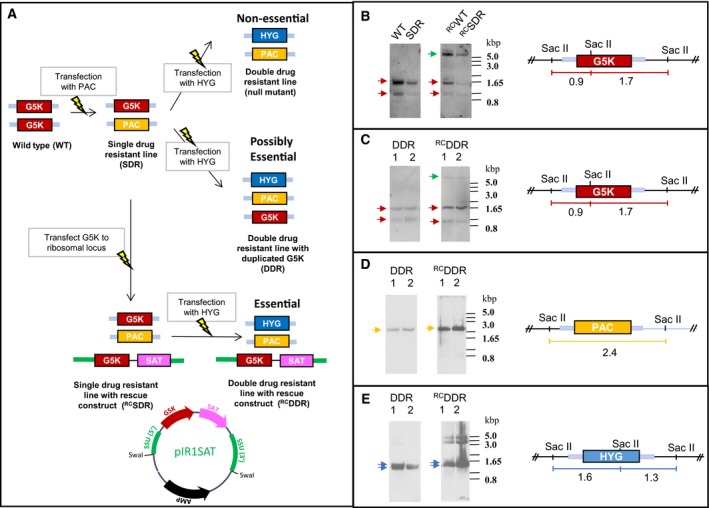
Assessing the essentiality of G5K. (A) Schematic representation of the gene replacement strategy and possible outcomes. The light blue bars represent the 5′‐ and 3′‐ UTRs flanking the G5K locus used for gene replacement by homologous recombination. A map of the rescue construct pIR1SAT_*LdG5K* is included showing the Swa I restriction sites used to linearise the construct targeted for insertion into the (ectopic) ribosomal DNA locus. (B–E) Genotypic analysis of transgenic clones for WT, SDR, DDR and their corresponding rescue constructs (^RC^WT,^RC^SDR and ^RC^DDR). gDNA samples were digested with Sac II and Southern blots prepared as described in Experimental procedures. Blots were sequentially probed and stripped with the following: LdG5K (B, C); puromycin *N*‐acetyl transferase (D) and hygromycin phosphotransferase (E). Coloured arrows indicate that gene replacement had occurred at the G5K chromosomal locus. Presence of the rescue construct in WT, SDR and DDR clones is highlighted by a green arrow.

To assess the possible role of G5K in the leishmania life cycle, we sought to generate a proline auxotroph by gene replacement. For a nonessential gene this is normally straightforward requiring two rounds of gene replacement with drug selectable markers to produce a null mutant as shown in the schematic diagram (Fig. 7A). In the case of an essential gene, gene duplication takes place, sometimes involving partial or complete chromosomal duplication; alternatively, the gene of interest is retained at the correct locus and the drug selectable marker is inserted elsewhere in the genome. Such genomic rearrangements are suggestive, but not conclusive evidence that a gene is essential. Addition of a ‘rescue copy’ of the gene of interest either expressed on a plasmid or targeted to a different genomic locus (e.g. ribosomal DNA locus) frequently allows replacement of both chromosomal copies, although this can also fail for technical reasons or the locus being refractory to genetic manipulation 28.

It is important to note that, although leishmania are aneuploid, the G5K‐bearing chromosome 26 is diploid in all species 29, 30. Restriction enzyme mapping confirmed that G5K is present as a single copy per haploid genome in the LdBOB strain used here. Single drug‐resistant lines (SDR) were obtained by transfection with the *PAC* gene replacement construct followed by selection for puromycin resistance in either WT leishmania or in WT cells previously transfected with the overexpression rescue cassette for G5K (^RC^WT). Resistant lines were obtained bearing the correct replacement of one G5K allele to produce a SDR and ^RC^SDR clonal line (Fig. 7B). Attempts to delete the second allele by transfection with the *HYG* construct in the SDR clone to produce a double knockout lacking G5K were unsuccessful (Fig. 7C). Although Southern blot analysis confirmed that PAC and HYG had been correctly integrated at the G5K locus (Fig. 7D,E), a copy of the endogenous G5K was also retained at the correct genomic locus (Fig. 7C). Transfection of the clone bearing the ectopic rescue construct (^RC^DDR) with *HYG* also failed to generate a chromosomal null mutant (Fig. 7C). The detection of two higher bands (5–4 kbp range) with the HYG marker as well as a doublet band potentially representing the correct size 1.6 and 1.3 kb fragments indicates correct insertion at the G5K locus and elsewhere in the genome (Fig. 7E). There was little difference in growth rates for WT, DDR and the equivalent rescue clonal lines with doubling times of 8.41 ± 0.07; 8.54 ± 0.05; 9.09 ± 0.03 and 9.85 ± 0.01 h respectively. To establish that the overexpressing lines were producing functional protein that would act as a rescue when both allelic copies were deleted, extracts of WT and ^RC^WT lines were assayed for G5K activity. In the ^RC^WT extract a net G5K activity of 34.7 ± 0.9 nmol·min^−1^·mg^−1^ was obtained against a background ATPase activity of 12.9 ± 1.8 nmol·min^−1^·mg^−1^. No net G5K activity could be detected for the WT extract due to the high background ATPase activity. However, addition of recombinant LdG5K to the WT extracts was readily measurable indicating that the extracts did not contain an inhibitor of the reaction. Thus, the failure to create a chromosomal null mutant cannot be ascribed to failure of G5K expression, but could be due to other technical reasons. Together, the results obtained here are suggestive, but not conclusive that G5K is essential for growth and survival of the parasite.

## Discussion

This study provides definitive evidence that the protein encoded by LdBPK_262740.1 is a *bone fide* G5K (http://www.chem.qmul.ac.uk/iubmb/enzyme/EC2/7/2/11.html) and not an aspartate kinase (http://www.chem.qmul.ac.uk/iubmb/enzyme/EC2/7/2/4.html). The kinetic parameters are broadly equivalent to the few prokaryotic G5Ks that have been studied in any detail (Table 1). Catalytic processes for microbial G5K's and plant P5CS enzymes are regulated through proline feedback inhibition. Proline, the end product of the pathway, induces the parasite enzyme to switch from hyperbolic to sigmoidal behaviour with respect to glutamate, with changes in the *S*_0.5_ for glutamate, Hill coefficient and *k*_cat_ similar to those reported for *E. coli* G5K 27. However, unlike the *E. coli* enzyme 23 the tetrameric structure of LdG5K is not affected by proline possibly due to the lack of a PUA domain. In *E. coli* G5K the addition of proline shifts the oligomeric state from tetramer (a dimer of dimers in crystallographic studies 31) to a possible dodecamer (trimer of tetramers) at infinite proline concentration 23. Deletion of the PUA domain (absent in LdG5K) abolishes this aggregation, but not the feedback inhibition by proline. Molecular modelling and site‐directed mutagenesis studies on the *E. coli* and *Campylobacter jejuni* G5Ks predict a proline‐binding pocket adjacent to the substrate binding site involving Asn34 and Asp 137 23, residues that are conserved in LdG5K (Fig. 3). The structure‐activity relationship of proline analogues determined here agree well with, and extend, the previous study 23 and emphasise the specificity of this binding pocket. Based on the estimated intracellular concentrations of glutamate (14 mm) and proline (3 mm) of promastigotes grown in amino acid replete medium 32, our studies predict that the enzyme will be essentially inactive under these nutrient‐rich conditions (Fig. 6A). Thus, the regulatory parameters determined here appear to be physiologically relevant to the intact cell, supporting the observation that biosynthesis only occurs under conditions of amino acid starvation 14. Unlike the long isoform of mammalian P5CS 33, which is involved in arginine biosynthesis, citrulline and ornithine do not inhibit LdG5K. This is consistent with the fact that leishmania are auxotrophic for arginine when cultured in the presence of glucose 32 and lack genes for two key pathway enzymes linking proline biosynthesis with ornithine / arginine metabolism (ornithine aminotransferase and ornithine deiminase).

Metabolism of proline in leishmania has similarities and differences with its trypanosomatid cousins, the African trypanosome, *Trypanosoma brucei* and the South American trypanosome, *Trypanosoma cruzi*. While all three species are capable of using proline as an energy source, converting proline into glutamate and subsequently into tricarboxylic acid intermediates 6, 18, 34, 35, metabolic labelling studies show that only leishmania is capable of synthesising proline *de novo* from either glutamate or glucose 14, 18, 34, 36. There is an absolute requirement for proline to support growth of *T. brucei* procyclic forms 18, whereas this is not the case for several *Leishmania* spp. 32. Given the lack of bioinformatic or biochemical evidence for alternative routes for proline biosynthesis from ornithine (via ornithine δ‐aminotransferase or ornithine cyclodeaminase) 37 this suggests that the G5K pathway is necessary and sufficient for growth of promastigotes in media lacking proline.

In addition to proline's role as a protein building block, as a major energy source and as a source of nitrogen, this imino acid has been implicated in response to various forms of cellular stress. Intracellular accumulation of proline is a common phenomenon in response to environmental stress in bacteria, protozoa, algae, plant and marine invertebrates (for a review see 36, 38). In other organisms proline has been shown to function as an osmoprotectant, as a small molecule chaperone preventing protein aggregation, as a metal chelator and as a scavenger of reactive oxygen species (ROS), specifically the hydroxyl radical and singlet oxygen 38. Proline is also proposed to help maintain the GSH thiol redox balance by scavenging ROS and by stabilising key antioxidant enzymes.

These possible roles for proline have not been extensively studied in the trypanosomatids. Proline, along with glutamate and alanine, have been implicated in responses to osmotic stress 39, 40 and this may be of biological relevance to these parasites’ survival in the insect gut. In *T. cruzi* proline is reported to modulate resistance to ROS and resistance to trypanocidal drugs through a novel d,l‐proline transporter 41. *T. cruzi* also possesses an intracellular proline racemase and a secreted proline racemase that acts as B‐cell mitogen modulating the immune response 42. However, the leishmania genome does not contain a proline racemase.

One specific role for proline in leishmania is in response to purine starvation 43. Leishmania are purine auxotrophs and lack the ability to synthesise purines *de novo*. Thus, they are entirely dependent on an exogenous supply from the extracellular medium. Purine starvation not only markedly upregulates purine transporters and purine salvage enzymes but also upregulates proline biosynthetic pathway and downregulates proline degradation, resulting in a 6‐ to 10‐fold increase in intracellular proline concentration 43. The role of proline in this stress response is not understood, but could act to augment the increased levels of proteins involved in protein folding/protein stability, as well as key trypanothione‐dependent antioxidant enzymes observed 43.

One key question is why do *Leishmania* spp. have this *de novo* biosynthetic pathway, whereas *T. brucei* and *T. cruzi* do not? One possibility concerns the different parasite locations in their mammalian hosts. *T. brucei* resides extracellularly in the blood, lymph and interstitial fluid, whereas *T. cruzi* resides intracellularly in the cytosol of many different cell types in many different tissues. Thus, both of these organisms should have ready access to proline and therefore have no requirement for *de novo* biosynthesis. In contrast, leishmania parasites reside within an acidic phagolysosomal compartment in macrophages – the parasitophorous vacuole (PV). Information on the availability of nutrients in the PV is lacking and can only be inferred from the nutritional requirements of the parasites. Thus, purines, haem, vitamins, iron and essential amino acids must be available in the PV in sufficient amounts to sustain growth 44. Studies with *Salmonella* auxotrophs suggest that the macrophage acidic compartment in which they reside is nutrient poor for purine, pyrimidine, aromatic amino acids, histidine and proline 45, 46, 47. Notably, *Salmonella enterica* lacking G5K (*ProB*) are defective for survival *in vitro* in macrophages and *in vivo* in mice 46. A related study in *Mycobacterium tuberculosis* showed that a proline auxotroph (*proC*, Δ^1^‐pyrolline 5‐carboxylate reductase) displays attenuated virulence both in bone marrow‐derived macrophages and in mice 48. While both of these studies indicate that proline is limiting in these specialised macrophage phagolysosomal compartments, it may not apply to the leishmania PV, which is known to be in contact with the external medium via endocytosis 49.

To assess the possible role of G5K in the leishmania life cycle, we sought to generate a proline auxotroph by gene replacement. Despite the presence of proline in the medium that should serve as a nutritional rescue, it was not possible to create a G5K null mutant. Instead, both drug selection markers were correctly inserted at the G5K locus, with retention of an additional copy of G5K. This result is suggestive, but not conclusive, that G5K is essential. Additional evidence of essentiality can be obtained using a genetic rescue strategy that should allow direct gene replacement without additional chromosomal alterations. In our case, multiple attempts to create a chromosomal G5K null with an episomal rescue construct in the ribosomal locus were unsuccessful even in the presence of proline (0.35 mm) in the growth medium. Further work is required to assess whether G5K has an additional structural or enzymatic function that remains to be elucidated.

A second possible unique function relates to a class of drugs used to treat leishmaniasis, namely, the pentavalent antimonials, sodium stibogluconate (pentostam) and meglumine antimonate (glucantime). These metalloid‐sugar complexes are thought to be activated by reduction of Sb^V^ to the trivalent (Sb^III^) form in the host macrophage, the intracellular amastigote or possibly both 3. The mode of action of Sb^III^ is not fully understood, but is likely to include inhibition of key dithiol antioxidant proteins such as trypanothione reductase and tryparedoxin peroxidase with perturbation of thiol‐redox homeostasis and thiol‐buffering capacity 50, 51, 52, 53. Resistance to antimonial compounds in Bihar State, India is now so widespread that antimonials are no longer recommended as front‐line treatment. One theory why this has occurred in India, but not in other geographic regions, relates to the metalloid, arsenic. The hypothesis is that the human population living in parts of Bihar are exposed to high levels of arsenic in drinking water and, in infected patients, arsenic selects for parasite resistance to trivalent arsenic and concomitant resistance to antimonials 54. Evidence to support this theory has been obtained *in vitro* 55, 56 and in animal models 57, although a retrospective clinical epidemiological study proved to be insufficiently powered to provide a definitive answer to this question 58. It is noteworthy that arsenic‐contaminated soil has a harmful effect on plant growth through perturbation of numerous metabolic pathways and other biological functions 59 and antimony‐contaminated soil induces similar adverse effects on growth 60. Strikingly, plants exposed to either inorganic arsenic or antimony respond by increasing their intracellular proline content 61, 62, 63. In leishmania, no changes in expression of G5K, glutamate‐5‐semi‐aldehyde dehydrogenase or pyrroline‐5‐carboxylate reductase have been noted in whole‐genome sequencing, proteomics or gene expression studies comparing drug‐sensitive and drug‐resistant parasites 64, 65, 66, 67, 68. However, a metabolomic study noted elevated glutamate and proline in antimony‐resistant cells compared with susceptible *Leishmania infantum* promastigotes 69. The mechanism by which glutamate or proline convey resistance is not understood: both amino acids are precursors for the antioxidant metabolites glutathione and trypanothione and proline could act as a scavenger of ROS induced by Sb^III^ 70. Whether these changes in amino acid content occur in antimony‐resistant clinical isolates has not been studied.

In conclusion, this study confirms the key putative enzyme in proline biosynthesis is indeed a classical G5K. G5K lacks aspartokinase activity leaving the question of how parasites synthesise threonine unanswered. Further work is required to establish the functional roles of proline in this parasite. In particular, is G5K a potential drug target in the intracellular amastigote stage of the life cycle?

## Experimental procedures

### Chemicals and reagents

Chemicals and reagents used in this study were all of the highest purity which are commercially available. All substrates, cofactors and enzymes were products from Sigma Aldrich (Gillingham, UK).

### *Leishmania donovani* promastigote cell culture maintenance

*Leishmania donovani* LdBOB promastigotes (derived from MHOM/SD/62/1S‐CL2D) were routinely passaged in modified M199 medium with 10% fetal calf serum as previously described 71.

### Cloning and generation of transgenic cell lines

All constructs made were prepared and sequenced for electroporation using QIAprep Miniprep Plasmid Kit (Qiagen, Venlo, the Netherlands). Primers used in this study to generate the overexpression and knockout constructs (Table 3) are based on the GeneDB sequences for *L. infantum* G5K (LinJ.26.2740).

**Table 3:** Primers used for knockdown, knockout and rescue constructs. Upper case letters refer to gene sequences and enzyme restriction sites are underlined

Primer name	Sequence
ORF‐BamHI forward	5′‐ggatccaATGGCGGACATCTTGAAG‐3′
ORF‐BamHI reverse	5′‐ggatccTCAAGATGAAACTCTCGA‐3′
5′UTR‐NotI forward	5′‐ataagaatgcggccgcCGCTAATGATTACACTAC‐3′
5′UTR‐HindIII/PmeI reverse	5′‐gtttaaacttacggaccgtcaagcttGCTTGATTCCGCGTGGAC‐3′
3′UTR‐PmeI/BamHI forward	5′‐gacggtccgtaagtttaaacggatccAGCGCTTAGGGATGTCAC‐3′
3′UTR‐NotI reverse	5′‐ataagtaagcggccgcGTTCGATACCGTTTTGAGA‐3′
*G5K*‐BamHI forward	5′‐gcgcggatccATGGCGGACATCTTGAAGT‐3′
*G5K*‐EcoRI forward	5′‐gcgcgaattcTCAAGATGAAACTCTCGAAT‐3′

For the gene replacement cassettes, the 5′ and 3′ UTRs directly adjacent to the ORF were amplified using 5′UTR‐*Not*I forward, 5′UTR‐HindIII/PmeI reverse and 3′UTR‐Pme I/BamHI forward, 3′UTR‐*Not*I reverse respectively. Using the following conditions: 95 °C for 1 min; 55 °C for 2.5 min; 72 °C for 3 min for 30 cycles, PCR products were used together in a further overlapping extension PCR to yield a product containing the 5′‐UTR linked to the 3′‐UTR via a short HindIII – BamHI linker region with a NotI site at each end. (Table 3). This product was subsequently ligated into the NotI site of pGEM‐5Zf(+) vector (Promega, Southampton, UK). The selectable drug resistance genes puromycin *N*‐acetyl transferase (*PAC*) and hygromycin phosphotransferase (*HYG*) were introduced into this vector via the HindIII and BamHI within the linker region cloning sites and sequenced.

To generate a recovery construct expressing LdG5K, pIR1SAT_*LdG5K*, the G5K ORF was amplified from *L. donovani* genomic DNA using oeG5K forward and reverse primers (Table 3) and subsequently cloned into the integrating stable expression vector pIR1SAT 72. For recombinant expression in *E. coli*, LdG5K was amplified from the pIR1SAT_*LdG5K* construct, with G5K forward and reverse primers (Table 3), subsequently cloned into a modified pGEX‐6P‐1 vector (Amersham Biosciences, Buckinghamshire, UK) containing in‐frame N‐terminal His‐GST tag, using the BamHI and the EcoRI restriction sites. All the constructs generated were verified by DNA sequencing (http://www.dnaseq.co.uk).

### Generation of *L. donovani* promastigote G5K knockout and overexpresser cells

DNA constructs were prepared for electroporation into LdBOB promastigotes and cloned with all procedures performed as previously described 73. Briefly, parasites were electroporated using Human T‐cell Nucleofactor with programme V‐033. Post‐transfection, cells were allowed to rest for 16–24 h prior to appropriate selection for PAC (20 μg·mL^−1^ puromycin) or HYG (50 μg·mL^−1^ hygromycin). Cloned cell lines were generated by limiting dilution and maintained in the presence of selective drugs. To generate WT and SDR lines overexpressing LdG5K, the pIR1SAT_*LdG5K* recovery construct was linearised by digestion with SwaI and electroporated into cells with selection for expression of streptothricin acetyltransferase (SAT) with 100 μg nourseothricin·mL^−1^.

### Genotypic analysis

DNA was prepared for Southern blot analysis from WT and KO cells. WT DNA (5 μg) was digested various restriction and resolved on a 0.8% agarose gel. The DNA was subsequently transferred onto nitrocellulose and UV cross‐linked. The blots were hybridised with probes specific for *G5K* ORF and 5′ UTR. The probes were labelled with fluorescein–dUTP by DIG labelling kit (Roche, Welwyn Garden City, UK) and the blots processed as previously described 74.

### Recombinant expression and purification of LdG5K

Recombinant pHis‐GST:*Ld*G5K was expressed in *E. coli* strain C41(DE3) pLysS. Transformed cells were cultured in auto‐induction medium (LB medium supplemented with 0.5 g·L^−1^ glucose and 2 g·L^−1^ α‐lactose) plus 100 μg·mL^−1^ ampicillin at 37 °C with shaking at 200 r.p.m. for 2 h and the temperature was reduced to 18 °C overnight. Uninduced cells were grown in medium lacking glucose and α‐lactose. Soluble protein was purified from clarified lysate on glutathione (GST) resin. Briefly, cells were harvested by resuspending in lysis buffer [PBS/0.25 m NaCl/5% (v/v) glycerol] supplemented with EDTA‐free complete protease inhibitor cocktail (Roche) and lysed using a continuous cell disruptor (Constant Systems, Daventry, UK) at 30 000 psi. Clarified lysates (centrifuged at 45 000 ***g*** for 45 min, 4 °C) were applied to GST resin (GE Healthcare, Little Chalfont, UK), pre‐equilibrated in lysis buffer. The lysate was batch bound at 4 °C for 2 h with subsequent washing prior to on‐column cleavage by PreScission protease for 24 h according to manufacturer's instructions. Protein fractions were analysed by SDS/PAGE using a NuPAGE Novex 4–12% Bis‐Tris gel (Life Technologies, Paisley, UK) and visualised for purity by Coomassie Brilliant Blue staining. Prepared enzyme was buffer exchanged and concentrated using 3 MWCO filtration unit (Vivaspin 20; Millipore, Watford, UK) and stored in 50 mm HEPES pH 7.5 containing 20% (v/v) glycerol and 0.0005% (w/v) Na azide as snap‐frozen aliquots at −80 °C. The sequence and identity of the purified protein was verified by tryptic mass fingerprinting with 79% sequence coverage (Proteomic and Mass Spectrometry facility, University of Dundee). Proteins were analysed by SDS/PAGE using 4–15% Bis‐Tris NuPAGE polyacrylamide gels (Invitrogen, Life Sciences, Paisley, UK) and 1× MES running buffer according to manufacturer's instructions. Protein concentration was measured spectrophotometrically at 595 nm using the Bradford reagent (Bio‐Rad, Watford, UK) and BSA as a standard 75.

### Analytical gel filtration

The native confirmation of LdG5K was confirmed by size exclusion chromatography using a Superdex 200 H/R 10/30 column fitted onto an AKTA FPLC system (both from GE Healthcare) with a buffer comprising 50 mm Tris‐HCl, pH 7.2 and 0.15 m NaCl running at 0.5 mL·min^−1^ at room temperature. Gel filtration protein standards (Bio‐Rad) used were as follows: vitamin B12 (1350 Da); horse myoglobin (17 000 Da); chicken ovalbumin (44 000 Da); bovine γ‐globulin (158 000 Da) and bovine thyroglobulin (670 000 Da). The void volume was 8.3 mL. The equation for estimating the molecular mass was derived from plots of *V*_e_/*V*_o_ against log MW of the standards.

### Kinetic characterisation of recombinant LdG5K and clarified *L. donovani* lysates

A continuous spectrophotometric enzyme assay was developed to follow the conversion of ATP into ADP using a coupled enzyme system utilising pyruvate kinase (PK) and lactate dehydrogenase (LDH; Fig. 4). Both coupling enzymes were present in excess and G5K activity was measured by the consumption of NADH at 340 nm. Standard assay conditions contained the following in a 1 mL reaction mix: 0.1 m imidazole, pH 7.4; 1 mm DTT; 5 mm ATP; 5 mm MgCl_2_; 0.1 mg·mL^−1^ BSA; 50 U PK/LDH; 1 mm phosphoenolpyruvate; 250 mm NADH; 0.75 μg LdG5K and the reaction was initiated with 100 mm Na glutamate. The assay components were pre‐equilibrated at 25 °C for 5 min prior to initiation with the substrate. When ATP was varied, 150 mm Na glutamate and 5 mm MgCl_2_ were kept in excess of ATP. When glutamate was varied 5 mm ATP and 5 mm MgCl_2_ were kept constant. Activity was calculated from the initial linear rate with the extinction coefficient of NADH, 6220 m ^−1^·cm^−1^ 76. All data were analysed by nonlinear regression using GraFit and fitted to the Michaelis–Menten equation, except for determining the free Mg concentration requirement which was fitted to a high substrate inhibition equation:

$$\nu =\frac{V_{\max}}{1+\frac{K_{m}}{S}+\frac{S}{K_{i}^{s}}}$$

In the presence of proline the enzyme displayed sigmoidal kinetics with respect to glutamate and data were fitted by nonlinear regression to the Hill equation describing allosteric behaviour:

$$\nu =\frac{V_{\max}{\left[S\right]}^{n}}{K^{n}+{\left[S\right]}^{n}},$$

where *n* = Hill coefficient.

Log‐phase cultures of *L. donovani* WT and WT‐overexpressing G5K (1 × 10^7^ cells·mL^−1^) were harvested by centrifugation (800 ***g***, 10 min, 4 °C) and washed twice with ice‐cold PBS containing cOmplete™, EDTA‐free protease inhibitor cocktail (Roche, Basel, Switzerland). Clarified lysates were prepared as previously described 74. Briefly, cells were osmotically lysed with ice‐cold distilled water (containing cOmplete™, EDTA‐free protease inhibitors), frozen and thawed three times in liquid nitrogen (a biosafety procedure) and finally resuspended in an equal volume of 2× assay buffer (0.2 m imidazole, pH7.4; 2 mm DTT). The lysates were buffer exchanged into 1× assay buffer to remove cofactors and substrates prior to determining protein content.

### Inhibitors

Initially single‐point assays of a set of proline analogues were tested at 1 mm concentration using the coupled assay described above. Any analogue which resulted in > 70% inhibition in LdG5K activity was retested under varying concentrations to determine their IC_50_ value. All measurements were done in triplicate.

### Phylogenetic analysis

The phylogenetic tree was constructed from a highly conserved region of deduced G5K and P5CS amino acid sequences. The evolutionary history was inferred using the Neighbour‐Joining method 77. The optimal tree with the sum of branch length = 7.43079248 is shown. The tree is drawn to scale, with branch lengths in the same units as those of the evolutionary distances used to infer the phylogenetic tree. The evolutionary distances were computed using the Poisson correction method 78 and are in the units of the number of amino acid substitutions per site. The analysis involved 24 amino acid sequences. All positions containing gaps and missing data were eliminated. There were a total of 203 positions in the final dataset. Evolutionary analyses were conducted in mega7 79.

## Conflicts of interest

The authors declare no real or perceived conflicts of interest.

## Author contributions

AHF conceived the project and NS, HBO and AHF designed the experiments. NS performed the experiments. All authors analysed the data and contributed to the manuscript.
